# Comparison of Prognostic Values of Seven Immune Indexes in Advanced Non-Small-Cell Lung Cancer Treated with Nivolumab: How Effective Can They Be Regarding Our Treatment Decisions?

**DOI:** 10.3390/medicina60111792

**Published:** 2024-11-01

**Authors:** Arife Ulas, Beyza Temel, Fahriye Tugba Kos

**Affiliations:** 1 Department of Medical Oncology, University of Health Sciences, Bursa City Education and Research Hospital, 16059 Bursa, Turkey; 2 Department of Internal Medicine, University of Health Sciences, Bursa City Education and Research Hospital, 16059 Bursa, Turkey; beyzakibar741@gmail.com; 3 Department of Medical Oncology, University of Health Sciences, Ankara City Education and Research Hospital, 06290 Ankara, Turkey; tugbasan@yahoo.com

**Keywords:** pan-immune inflammation value (PIV), systemic immune inflammation value (SII), neutrophil-to-lymphocyte ratio (NLR), platelet-to-lymphocyte ratio (PLR), monocyte-to-lymphocyte ratio (MLR), derived neutrophil-to-lymphocyte ratio (d-NLR), prognostic nutritional index (PNI), nivolumab

## Abstract

*Background and Objectives*: In this study, we evaluated the impact of seven immune indexes on treatment response and survival outcomes in advanced non-small-cell lung cancer (NSCLC) patients receiving second-line and subsequent nivolumab treatment under real-life conditions. *Materials and Methods*: The pan-immune inflammation value (PIV), systemic immune inflammation value (SII), neutrophil-to-lymphocyte ratio (NLR), platelet-to-lymphocyte ratio (PLR), monocyte-to-lymphocyte ratio (MLR), derived neutrophil-to-lymphocyte ratio (d-NLR), and prognostic nutritional index (PNI) were calculated. All immune indexes were classified as low and high based on cut-off values. Kaplan–Meier and Cox hazard models were used for survival analysis. *Results*: The median follow-up was 22.0 months (6.0–96.0). The median overall survival (OS) was 30.0 months and the median progression-free survival (PFS) was 7.0 months. In the univariate analysis, comorbidity (*p* = 0.03) and nivolumab use for more than eight cycles (*p* < 0.0001) were associated with an increase in PFS, while smoking history (*p* < 0.005) and d-NLR (*p* < 0.05) were more effective regarding OS. Patients who received more than eight cycles of nivolumab had longer median PFS (4 vs. 19 months, *p* < 0.001) and OS (23 vs. 43 months, *p* < 0.001). We found longer median OS in the PLR (45.7 vs. 75.4 months; *p* = 0.05), PIV (53.0 vs. 66.4 months; *p* = 0.19), SII (50.0 vs. 71.9 vs. months, *p* = 0.19), and NLR (49.9 vs. 74.55 months, *p* = 0.10) indexes in nivolumab long-term users (high vs. low groups, respectively). In short-term users of nivolumab, only d-NLR median OS (high vs. low, 19 vs. 75.2 months, *p* = 0.07) was different. Complete and partial response rates to nivolumab treatment were higher in the PNI-high group (*p* = 0.04). *Conclusions*: In these real-life data, we determined that the PLR, PIV, SII, and NLR indexes were effective in the prognosis of patients who received PD1 inhibitor nivolumab for a long time, and the d-NLR index was effective in those who developed progression in a short time. We found that the PNI was effective in patients who responded well to ICI treatment.

## 1. Introduction

Lung cancer constitutes a significant portion of cancer-related deaths. When GLOBOCAN 2022 data are evaluated, lung cancer ranks first among all cancers in terms of both incidence (12.4%) and mortality (18.7%) [1]. Thanks to recent advances in molecular biology, the use of on-target therapies, immunotherapy (ICI), and immunotherapy + chemotherapy (CT) combinations have significantly improved survival in non-small-cell lung cancer. The greatest success in cancer treatment in the last decade is undoubtedly the discovery of T cell-targeted immunomodulators that inhibit immune checkpoints such as Cytotoxic T-Lymphocyte Antigen 4 (CTLA-4), programmed death receptor-1 (PD-1), and programmed death ligand 1 (PD-L1) [2].

Cancer immunotherapy aims to utilize the ability of the immune system to recognize and destroy cancer cells. PD-1, a type I transmembrane protein, is found in naturally activated T cells, B cells, natural killer cells, macrophages, dendritic cells, and monocytes. As a result of its association with PD-L1 on the surface of the antigen-presenting cell (APC), the T cell response and anti-apoptotic processes are inhibited [3]. Nivolumab, a PD-1 inhibitor developed to prevent the immune escape mechanism, showed a survival benefit in a comparative study with docetaxel in patients who developed progression after second-line platinum-based chemotherapy combinations (9.2 vs. 6 months, HR: 0.59) [4]. Pembrolizumab has been the standard of care in the first-line setting in aNSCLC with PD-L1 > 50% [5]. Currently, combinations of ICIs with chemotherapy and bevacizumab, as well as the anti-CTLA-4 agent ipilimumab and nivolumab regimens, have shown survival benefits compared to standard therapy and have emerged as first-line treatment options in advanced NSCLC [6].

Despite the survival benefit achieved with ICIs, only a proportion of patients achieve a durable clinical benefit from their ICI. To determine patients who respond well to immunotherapy, the identification of predictive or prognostic indexes is a critical aspect of clinical trials. Tumor mutational burden (TMB), PD-L1 expression, and immunoinflammatory markers are commonly used biomarkers to predict patients’ responses to ICIs [7]. These biomarkers have yielded contrasting results in some clinical trials: a considerable proportion of patients responded to these agents even in the absence of PD-L1 expression [4,8]. However, these markers are difficult to apply in the clinic due to their high cost and high expertise requirements. Therefore, several different markers of serum inflammation based on inflammation have been developed due to the interaction between systemic inflammation, the immune system, and immunotherapy. Inflammation-based scores such as neutrophil-to-lymphocyte ratio (NLR), monocyte-to-lymphocyte ratio (MLR), platelet-to-lymphocyte ratio (PLR), and derived neutrophil-to-lymphocyte ratio (d-NLR) have attracted attention due to their potential as prognostic indicators in various cancers [9,10]. Some systemic inflammation indicators such as high d-NLR and high PLR are related to poor response to both nivolumab and CT in NSCLC [11]. Systemic immune inflammation index (SII), pan-immune inflammation value (PIV), and prognostic nutritional index (PNI) results have been reported in patients with various cancers (lung cancer, gastrointestinal carcinomas, kidney cancer, breast cancer, malign melanoma, prostate cancer, etc.) [9,10,12,13]. PIV has been found to outperform other well-established immune biomarkers such as NLR and PLR in predicting patient outcomes [13]. Based on the relationship between cancer, nutrition, and inflammation, the PNI has been shown to predict the survival of patients with solid tumors including colorectal and NSCLC [14,15].

Studies over the last 20 years have shown that inflammatory immune cells are the main players in cancer-induced inflammation and have highlighted their role at different stages of the disease. In 1863, Virchow first proposed the hypothesis that cancer develops as a result of inflammation, the mechanism of which is not yet understood, and this hypothesis has formed the basis for understanding the relationship between cancer development and chronic inflammation [16]. The inflammatory response that occurs in the tumor microenvironment enables the recruitment of cell types such as macrophages, myeloid-derived suppressor cells, and mesenchymal stem cells. The reciprocal relationship between tumor cells and the surrounding stromal cells promotes tumor progression and creates a dynamic extracellular matrix suitable for invasive tumor cells [17]. In tumor-associated inflammation, peripheral blood cells such as neutrophils, platelets, and monocytes play a role in tumor progression by secreting pro-tumor cytokines that cause angiogenesis, invasion, and immunosuppression [18]. Lymphocytes, on the other hand, suppress tumor formation, and mediate the antitumor effects of CD8+ and CD4+ T cells in the tumor microenvironment [19]. Composite biomarkers such as NLR, PLR, MLR, and d-NLR include only two parameters, while SII consists of three parameters and PIV consists of four parameters. PIV is a four-cell biomarker of (PIV = Monocytes × SII) whose systemic immune inflammation index reflects the reformulated systemic and intra-tumoral inflammatory/immune system status [12].

Inflammation-based scores reflect many biological changes, including the host, tumor, and tumor microenvironment, and the impact of systemic inflammation on various aspects of patient physiology. In our daily practice, cost-effective, easy-to-calculate biomarkers that can be applied in a time that does not delay the treatment process are very important to predict patient survival and treatment response and facilitate treatment planning. Blood cell count is an easy-to-obtain, repeatable, and cost-effective index to represent the status of host immune inflammation.

In the present study, we assessed the prognostic impact of seven immune indexes based on immune inflammation in patients with NSCLC treated with second-line or single-agent nivolumab therapy using real-life data. In our data, instead of focusing on a single biomarker, we planned to evaluate seven immune indexes separately. We evaluated the contribution of binary parameters such as NLR, PLR, MLR, d-NLR, as well as PIV, a comprehensive marker including SII and four blood cells, and PNI scores, an indicator of nutrition and inflammation, in our daily practice in terms of individualized treatment planning.

## 2. Materials and Methods

### Study Design and Data Collection

This study was performed at the Department of Medical Oncology in the Bursa City Teaching and Research Hospital of the University of Health Sciences between October 2019 and March 2024. This research was a retrospective and single-center study. This research was approved by the local ethical committee and carried out in accordance with the ‘Declaration of Helsinki’.

A total of 436 NSCLC patients were reviewed. Patients were histologically diagnosed with NSCLC and staged using tumor–node–metastasis (TNM) criteria. The study included 104 patients with advanced NSCLC treated with nivolumab treatment (3 mg/kg, every 2 weeks) in the second line or beyond. Patients were excluded if they (1) had SCLC or did not have a primary diagnosis of lung cancer; (2) could not give detailed clinical data; (3) had missing laboratory data; (4) had clinical proof of active infection or inflammation; (5) had hematological disease and previous or ongoing autoimmune disorder; (6) had had pulmonary embolism, a severe acute myocardial infarction, or any cerebrovascular accident within one month; or had (7) oncogene-addicted malignancies (EGFR mutations, ALK mutations, ROS mutations, and Braf mutations).

Baseline characteristic data were collected, including age at treatment initiation, sex, performance status (Eastern Cooperative Oncology Group/ECOG), smoking history, tumor histology, stage at treatment initiation, prior lines of therapy, ICI therapy, and time on therapy. Data on the type of CT regimen and number of CT cycles were collected. Laboratory findings before nivolumab treatment (neutrophil count, platelet count, monocyte count, lymphocyte count, and albumin level) were recorded. The cases were staged according to the TNM system. Therapeutic responses were assessed using the Response Evaluation Criteria in Solid Tumors guidelines (version 1.1) every 10 ± 2 weeks. While assessing the treatment response, the RECIST criteria identified by the World Health Organization were taken into account. Overall survival (OS) was calculated from the beginning of nivolumab treatment to either the date of death for any reason or the date of the last follow-up. Progression-free survival (PFS) was calculated as the interval between the starting of nivolumab therapy and the progression of the disease, recurrence, or death for any reason.

The immune-inflammation-based indexes evaluated in our study were determined by the definitions and calculation methods in the literature. Neutrophil-to-lymphocyte ratio (NLR) was calculated as neutrophil count/lymphocyte, and platelet-to-lymphocyte ratio (PLR) was calculated as platelet count/lymphocyte count. Monocyte-to-lymphocyte ratio (MLR) was calculated as monocyte count/lymphocyte count, and derived neutrophil-to-lymphocyte ratio (d-NLR) was calculated as neutrophil count/(leucocytes count − neutrophil count). Pan-immune-inflammation value (PIV) was calculated as (neutrophil count × platelet count × monocyte count)/lymphocyte count. Systemic immune-inflammation value (SII) was calculated as (neutrophil count × platelet count)/lymphocyte count. The prognostic nutritional index (PNI) was measured using the following formula: 10 × serum albumin level (g/dL) + 0.005 × total lymphocyte count (per mm^3^). At baseline, the cut-off values for NLR, PLR, MLR, d-NLR, PIV, SII, and PNI as binary variables were truncated according to the receiver operating characteristic (ROC) curve.

## 3. Statistical Analysis

Median (interquartile range (IQR)) or mean ± standard deviation (SD) for continuous variables and percentages for categorical variables were used to describe the data. After the descriptive statistics, the Mann–Whitney U test was used to compare variables that did not show a normal distribution between the groups, and the Student T test was used to compare variables that showed a normal distribution. Chi-square and Fisher’s exact test were utilized to compare categorical data between the groups. Cut-off values were calculated by using AUC values with ROC analysis for prediction of mortality and recurrence for continuous variables. Patients were grouped as high- and low-risk according to these cut-off values. The sensitivity and specificity of the seven immune indexes for prognosis estimation were examined using a time-dependent (ROC) curve in Version 8.0.0, for windows. Kaplan–Meier estimates were used for survival analyses. Cox proportional hazard regression models were utilized for multivariate analysis of OS and PFS. To estimate independent variables for OS and PFS, regression models were constructed using the statistically significant variables in univariate survival analyses. A *p*-value less than 0.05 was accepted as statistically significant. All statistical analyses were carried out using SPSS 22.0 (IBM, Armonk, NY, USA). 

## 4. Results

### 4.1. Patients

In our study, the median age was 62 years (range 39–82) and 68.3% of the cases were younger than 65 years. In total, 90.4% were male and 55.8% were ECOG PS 1. Overall, 95% of patients were smokers and 53.8% had non-squamous carcinoma. In total, 61.5% of our patients had at least one comorbid condition. Overall, 19 patients had diabetes mellitus, 22 patients had hypertension, 21 patients had coronary artery disease, 19 patients had chronic obstructive pulmonary disease, and 18 patients had compensated chronic renal failure. Overall, 72 (69.3%) patients had undergone testing for tumor PD-L1 expression; 23.1% and 46.2% of patients tested positive and negative for PD-L1, respectively. In 12.5% of the patients, the PD-L1 expression rate was found to be greater than 50%. Among non-squamous patients, 12.5% were KRAS-mutated and 3.5% were found to have the Her2 mutation. The rate of patients who were metastatic at the time of diagnosis was 88.5%, and the sites of metastasis were bone, the adrenal glands, the brain, and the liver. The baseline characteristics of patients and the median value of immune scores are listed in Table 1.

### 4.2. Treatments

Overall, 86.6% of the patients received nivolumab in the second line and 13.4% received it after the second line. The median number of nivolumab cycles administered was 8 (range 4–48). In terms of response rates of nivolumab treatment, 4.8% had complete response, 35.6% had partial response, 18.3% had stable disease, and 33.7% had progression. For first-line treatment, platinum-based chemotherapy could be applied to 91.4% of all patients; 47.1% had received carboplatin plus paclitaxel, 9.6% had received cisplatin plus docetaxel, 26% had received platinum plus gemcitabine, and 5.8% had received platinum plus pemetrexed. In total, 8.6% of patients had received only single-agent gemcitabine chemotherapy. After first-line chemotherapy, complete response was obtained in 2.9% of patients, partial response in 58.7% of patients, progression in 27.9% of patients, and stable response in 8.7% of patients.

### 4.3. Analysis of Immune Indexes (PIV, SII, NLR, PLR, MLR, d-NLR, and PNI)

All immune indexes were categorized as low and high according to cut-off values. These were PIV-low (≤669.6), PIV-high (>669.6), SII-low (≤753.5), SII-high (>753.5), NLR-low (≤3.03), NLR-high (>3.03), PLR-low (≤166.9), PLR-high (>166.9), MLR-low (≤0.44), MLR-high (>0.44), d-NLR-low (≤1.85), d-NLR-high (>1.85), PNI-low (≤40.08), and PNI-high (>40.08).

ROC curve statistics revealed the following values: PIV (AUC 0.62; 95% CI: 0.51–0.73, *p* = 0.03, sensitivity 61%, specificity 58%), SII (AUC 0.60; 95% CI: 0.49–0.71, *p* = 0.04, sensitivity 61%, specificity 58%), NLR (AUC 0.61; 95% CI: 0.50–0.72, *p* = 0.04, sensitivity 66%, specificity 60%), PLR (AUC 0.62; 95% CI: 0.43–0.66, *p* = 0.03, sensitivity 61%, specificity 53%), MLR (AUC 0.63; 95% CI: 0.52–0.73, *p* = 0.02, sensitivity 61%, specificity 54%), d-NLR (AUC 0.61; 95% CI: 0.48–0.70, *p* = 0.04, sensitivity 62%, specificity 60%), and PNI (AUC 0.60; 95% CI: 0.35–0.69, *p* = 0.04, sensitivity 60%, specificity 56).

### 4.4. Relationship Between Clinicopathological Characteristics and Immune Indexes

There were differences between the two groups in terms of PIV between low and high immune inflammation indexes according to histopathologic subtypes (*p* = 0.05), NLR (*p* = 0.01), PLR (*p* = 0.05), MLR (*p* = 0.02), and d-NLR (*p* = 0.04). The rates of the high groups of these indexes were higher in the squamous group and were found to be PIV-high (61.7%), NLR-high (66.0%), PLR-high (66%), MLR-high (66%), and d-NLR-high (62.5%), respectively. In the non-squamous group, the rates of the low groups of these indexes were higher and were found to be PIV-low (56.4%), NLR-low (58.2%), PLR-low (52.7%), MLR-low (56.4%), and d-NLR-low (57.2%), respectively. Apart from the histopathological subtypes, in terms of comorbidity, there were comorbidities in patients with PIV-low (69.4%), while 28 (52.8%) patients with PIV-high had comorbidities. There was a difference in the distribution of the groups, but it was not statistically meaningful (*p* = 0.08). For BMI, 25 (51.0%) patients with the SII-low index had a low BMI, while 35 (66.0%) patients with the SII-high index had a low BMI (*p* = 0.09). When ECOG PS was examined, 23 (48.9%) patients in the MLR-low group had ECOG 1-2, while 40 (72.7%) patients in the MLR-high group had ECOG PS 1-2 (*p* = 0.01). There was no difference between the low and high immune inflammation indexes with smoking in PIV (*p* = 0.81), SII (*p* = 0.67), NLR (*p* = 0.67), PLR (*p* = 0.74), MLR (*p* = 0.67), d-NLR (*p* = 0.30), and PNI (*p* = 0.83). There was no difference between the low and high groups in terms of clinicopathologic features in other indexes.

### 4.5. Survival Analyses of Clinicopathological Characteristics and Immune Indexes

The median follow-up period was 22.0 months (6.0–96.0). Median OS for all patients was 30.0 months (95% CI: 22.0–37.0) and median PFS was 7.0 (95% CI: 5.1–8.8) months. Median OS in patients with de novo metastases was 25 months (95% CI: 23.3–26.6), while OS in patients with metastases during follow-up was 60.0 months (95% CI: 32.8–71.4) (*p* = 0.04). Median OS in women was 39 months, while it was 25.0 months in men (*p* = 0.07). Median PFS was shorter in non-smokers compared to smokers (5 vs. 9 months, *p* = 0.06). Median OS was shorter in non-smokers compared to smokers (24 vs. 31 months), but statistical significance was not achieved (*p* = 0.10). Median OS was 33 months (95% CI: 18.2–47.7) in non-squamous cases and 25 months (95% CI: 17.2–32.7) in squamous cases, but statistical significance was not achieved (*p* = 0.23).

Although there was a difference in median OS between the low and high groups for all immune inflammation indexes, statistical significance was not obtained. PIV (36 vs. 24 months, *p* = 0.16), SII (36 vs. 24 months, *p* = 0.15), NLR (36 vs. 25 months, *p* = 0.10), PLR (33 vs. 24 months, *p* = 0.19), MLR (38 vs. 25 months, *p* = 0.31), d-NLR (36 vs. 25 months, *p* = 0.29), and PNI (25 vs. 31 months, *p* = 0.85) did not achieve statistical significance. For median PFS, although there was a difference between the low and high groups in NLR (10 vs. 6 months, *p* = 0.54), MLR (9 vs. 6 months, *p* = 0.30), and d-NLR (9 vs. 6 months, *p* = 0.10), statistical significance was still not achieved.

**Figure 1:**
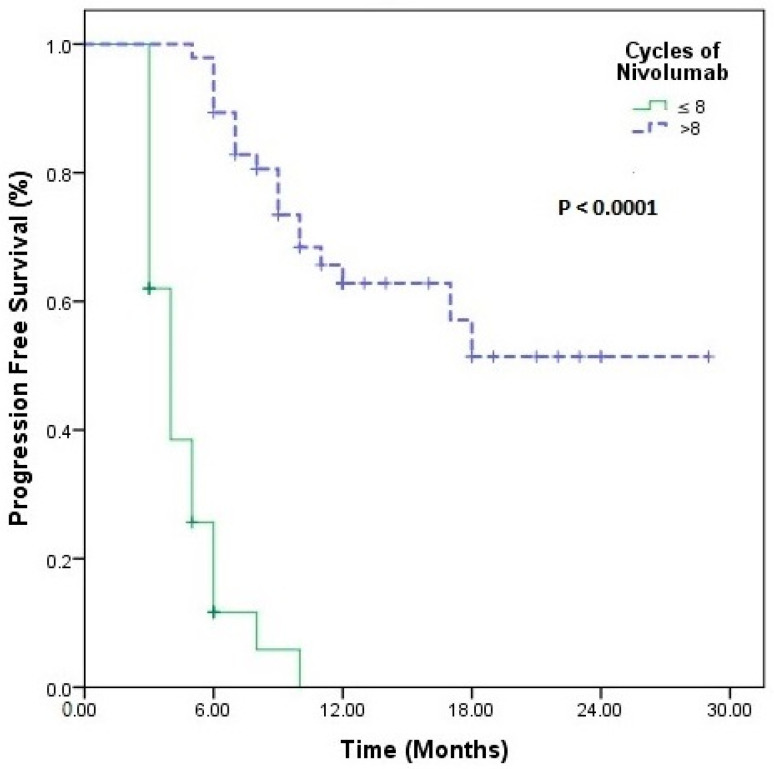
Progression-free survival survival in patients using nivolumab long term >8 cycles vs. ≤8 cycles nivolumab short term.

**Figure 2:**
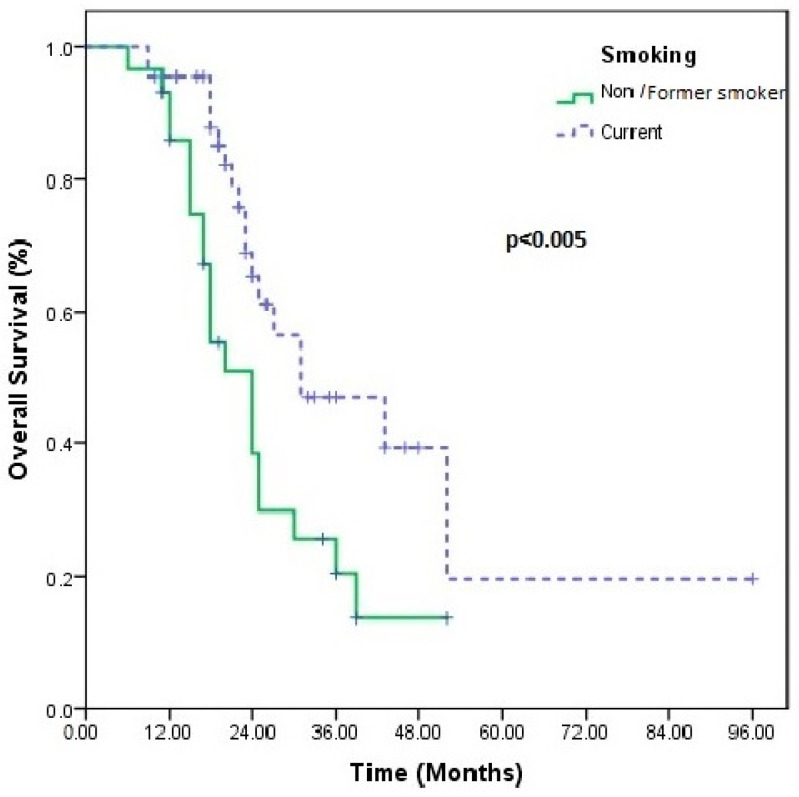
Overall survival in patients using non-smoker/former smoker vs. current smoker in univariate analyses.

In univariate analysis, only the presence of comorbidity (*p* = 0.03) and eight cycles of nivolumab (*p* < 0.0001) were associated with an increase in PFS, while smoking history (*p* < 0.005) and d-NLR (*p* < 0.05) were associated with an increase in OS (Table 2) (Figure 1, Figure 2). No statistical significance was obtained in the multivariate analysis.

**Figure 3:**
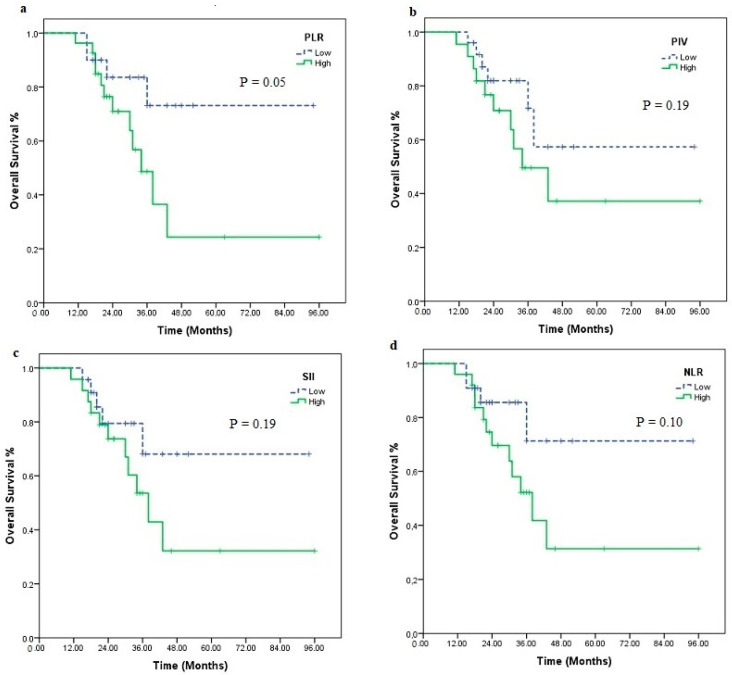
Kaplan–Meier estimates of overall survival in patients using nivolumab long term >8 cycles); overall survival according to PLR (**a**), PIV (**b**), SII (**c**), NLR (**d**).

**Figure 4:**
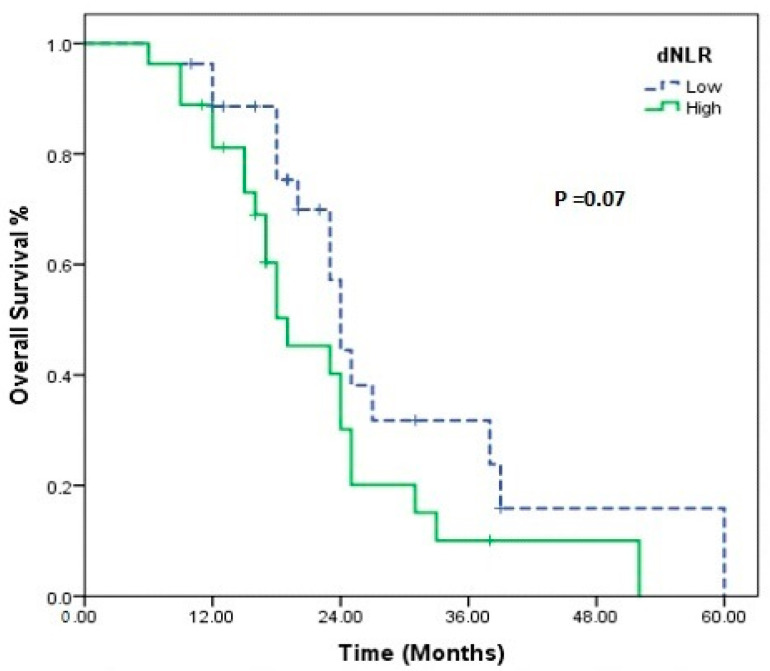
Kaplan–Meier estimate of overall in patients using nivolumab short term (≤8 cycles); overall survival according to, dNLR.

**Figure 5:**
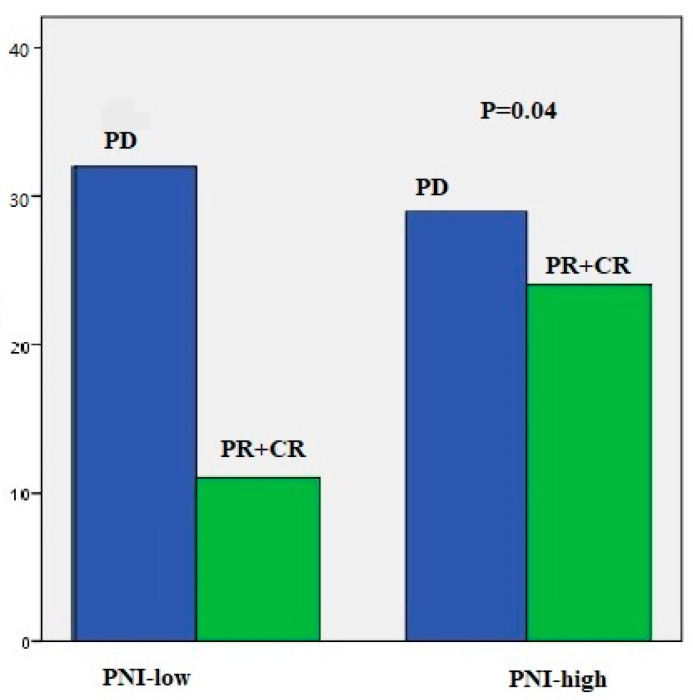
Treatment response rates in patients between PNI-low and PNI-high groups. CR, complete response; PR, partial response; PD, progressive disease.

When nivolumab treatment was categorized according to the median number of cycles (eight cycles), it was statistically meaningful for both PFS (4 vs. 19 months, *p* < 0.001) and OS (23 vs. 43 months, *p* < 0.001). Statistical significance was found for PLR (low vs. high, 75.4 vs. 45.7 months; *p* = 0.05) according to median OS in those receiving nivolumab > 8 cycles. There were also differences in PIV (low vs. high, 66.4 vs. 53.0 months; *p* = 0.19), SII (low vs. high, 71.9 vs. 50.0 months, *p* = 0.19), and NLR (low vs. high, 74.55 vs. 49.9 months, *p* = 0.10), although not statistically significant (Figure 3). In short-term nivolumab users, a difference was only found between d-NLR low and high groups (median OS: 24 vs. 19 months, *p* = 0.07) (Figure 4). In patients who achieved complete and partial response to nivolumab treatment, differences were observed in PIV (*p* = 0.52), SII (*p* = 0.52), NLR (*p* = 0.87), PLR (*p* = 0.48), MLR (*p* = 0.48), and d-NLR (*p* = 0.36), even though they were not statistically significant. In particular, in the PNI-high group, complete and partial response rates were high and showed a significant difference (*p* = 0.04) (Figure 5).

Nivolumab-related grade 2 or higher toxicities were observed in 56.7% of patients. These included endocrine dysfunction (14.6%) and fatigue (12.4%), with hypothyroidism being the most common. Other adverse reactions included weight loss (6.4%), rash (5.9%), pneumonitis (5.9%), GIS (3.8%), hepatotoxicity (4.8%), neurological findings (4.4%), and pericarditis/myocarditis (2.9%). Four patients discontinued treatment due to nivolumab-related toxicity (two patients due to neurological findings and two patients due to pneumonitis). There was no difference between the low and high immune inflammation indexes with immune-related adverse events in PIV (*p* = 0.30), SII (*p* = 0.30), NLR (*p* = 0.65), PLR (*p* = 0.27), MLR (*p* = 0.50), d-NLR (*p* = 0.39), and PNI (*p* = 0.26).

## 5. Discussion

Today, thanks to advances, we have learned that chronic inflammation and the avoidance of immune surveillance are essential features of cancer cells. Subsequently, ICIs, the shining star of the last decade, have been observed to significantly improve the therapeutic landscape of aNSCLC and prolong survival. However, in daily practice, it is crucial to determine the group of patients who respond to ICIs, especially in centers with high patient turnover.

Here, we calculated seven immune indexes based on the levels of neutrophils, lymphocytes, platelets, leukocytes, monocytes, and albumin in the peripheral blood of patients before immunotherapy treatment. We searched for an answer to the question of which immune index could be most easily and effectively used to better and quickly determine patients who would benefit from ICI therapy in our daily practice. Our results showed that these seven immune indexes, which are both easy and cost-effective, may be useful in predicting survival prognosis and treatment response in patients receiving long-term ICI.

In our study, in patients who received nivolumab treatment in the second line and beyond, regardless of pre-treatment PDL-1 IHC status, survival improved considerably in the group that benefited from treatment. In this study, we found a non-statistically significant difference in OS between the low- and high-immune index groups. However, we detected the effect of PIV, SII, PLR, and NLR in patients who received nivolumab for a long time, and d-NLR in those who received it for a short time. In non-squamous histology, median OS was longer in the lower groups of the PIV, NLR, PLR, and d-NLR indexes. On the other hand, PNI was more significant than other indexes in assessing treatment response in patients receiving ICIs.

Mediators and cellular effectors of inflammation are important components of the tumor microenvironment [20]. Genetic and epigenetic changes are required for tumor development and growth. An inflammatory component is present in the microenvironment of tumors that are epidemiologically unrelated to inflammation. An intrinsic (driven by genetic events that cause neoplasia) and extrinsic (driven by inflammatory conditions that predispose to cancer) pathway links inflammation and cancer [21]. The persistence of systemic inflammation is related to poor outcomes in many types of cancer, including NSCLC [9,10,11,12,13,14,15,22]. NLR has been suggested to better reflect the homeostasis between pro- and antitumor activity of the host immune system [23]. Neutrophils are the most abundant immune cells in circulation and their involvement in different stages of cancer progression supports the fact that neutrophil-containing indexes provide more meaningful results [24]. An increase in neutrophil numbers triggers the release of significant amounts of reactive oxygen and nitric oxide species, potentially leading to the deterioration of T cell function [25]. A high NLR and high absolute neutrophil count (ANC) have been related to worse prognosis and less response to conventional therapies in NSCLC [26]. The NLR has been studied in patients who have been treated with ICIs for different types of cancer, and a number of threshold values have been proposed [9,11,27]. On the other hand, not only the pretreatment NLR but also the variation in NLR throughout the treatment period has been shown to correlate with survival outcomes [28]. In 54 NSCLC patients treated with anti-PD-1, an NLR > 5 assessed at baseline and week 6 was significantly associated with lower RR, shorter PFS, and worse OS [29]. A high d-NLR has also been associated with poor survival [30] and hyperprogressive disease [31] in NSCLC patients treated with ICIs.

The link between the NLR and the outcome of cancer is likely to be due to tumor-associated changes in the immune system. The enzymes elastase, cathepsin G, and MMP9 secreted by neutrophils have been associated with progression in metastatic disease [32,33]. Neutrophil elastase directly induces tumor cell proliferation in both human and mouse lung adenocarcinomas by providing access to an endosomal compartment within tumor cells, where it cleaves insulin receptor substrate-1 (IRS-1). Immunoprecipitation studies showed that neutrophil elastase enhanced the interaction between phosphatidylinositol 3-kinase (PI3K) and the potent mitogen platelet-derived growth factor receptor (PDGFR), thereby tilting the PI3K axis towards tumor cell proliferation [34,35]. In addition, a high NLR reflects decreased lymphocyte-mediated immunity (with an altered CD4+ helper/CD8+ suppressor ratio) and increased production of inflammatory agents such as vascular endothelial growth factor (VEGF), which promote tumor growth [36].

In our study, both pre-treatment NLR-high (>3.03) and d-NLR-high (>1.85) were related to poorer PFS and OS. In univariate analysis, d-NLR was found to be more meaningful than other indexes in relation to OS. In patients who had received fewer than eight cycles of nivolumab, it was found that survival was worse in the group with a high d-NLR. This led us to ask whether d-NLR could be a more predictive immune score in patients with rapid progression under nivolumab. In addition, an important result was that complete and partial response rates were higher in patients with low-d-NLR. Furthermore, both high-NLR and high-d-NLR rates were higher in the squamous histology group. In addition to these indexes, high-PIV, high-PLR, and high-MLR rates were also more frequently detected in squamous histology. As a reflection of these results, OS in squamous histology was found to be shorter than in non-squamous, although not significantly. However, we know from meta-analyses that increased TMB, increased PD-L1 expression, tumor-infiltrating lymphocytes (TILs) in the tumor microenvironment (TME), chemokines, and organic driver alterations contribute to the response to ICIs in squamous NSCLC [37]. However, in some phase III randomized trials, ICIs were effective in both squamous and non-squamous types, but the results are variable. Nivolumab was found to be effective in both squamous and non-squamous types in the CheckMate 017 and CheckMate 057 studies in terms of PFS, OS, and ORR [38,39]. However, in the second-line study, ICIs were less effective in squamous-type than in non-squamous NSCLC in the Keynote 010 study (HR, 0.74 vs. 0.63) [7]. In the OAK study, OS results in patients given atezolizumab in the second line were similar in both histological types, independent of PDL-1 and histology [8]. In the Pacific study, non-squamous type, younger age, and female sex were shown to be favorable prognostic factors for OS [40].

Lately, an elevated PLR has been shown to be tightly associated with worse prognosis in a number of solid tumors [41]. Platelets cause epithelial–mesenchymal transition in tumor cells and contribute to metastasis. High platelet counts play an important role in inflammation, tissue remodeling, and tumor progression [42,43]. In particular, IL-6, an inflammatory cytokine, and vascular endothelial growth factor (VEGF) secreted by tumor cells have been shown to stimulate megakaryocyte differentiation and promote tumor growth [43,44,45]. In NSCLC patients treated predominantly with nivolumab, a higher PLR correlated with worse OS [46]. In our study, we also determined the prognostic effect of high-PLR (>166.9) and its association with shorter survival, especially in patients receiving long-term nivolumab treatment. In our study, PLR was efficient in patients with complete and partial response to nivolumab treatment. A meta-analysis reported that pretreatment PLR is a routine potential prognostic factor and may have a predictive role in patient survival [47].

In our study, PIV and SII (high-PIV > 669.6 and high-SII > 753.5), which include multiple parameters, were associated with shorter OS and PFS, albeit partially, in all of our patients. We also found that these two indexes affected survival in our patients receiving long-term nivolumab. In a major study, PIV was reported to have a greater relative effect on OS and PFS than NLR and SII [12]. PIV was found to outperform other well-established immune biomarkers such as NLR and PLR in predicting patient outcomes [13]. Another study in patients treated with ICI showed that a high PIV is related to poorer PFS and OS compared to SII, NLR, PLR, MLR, and d-NLR [48]. High PIV and high SII findings might be linked to worsening PFS and OS outcomes, decreased immunogenic lymphocyte counts, and higher inflammatory and immunosuppressive monocyte, platelet, and neutrophil counts. Although PIV may capture the complexity of the immune environment more comprehensively than individual blood cell parameters or other combined statistics, our study did not demonstrate superiority over other parameters.

In general, systemic inflammation and nutrition play crucial roles in cancer development, therapeutic effects, and cancer cachexia [49]. Similar to our result, previous trials have revealed that PNI based on albumin levels predicts survival of patients with solid cancers such as colorectal, esophageal, and NSCLC [14,50,51]. In our study, PNI showed significant differences compared with other immune indexes in patients with complete response and partial response to nivolumab treatment (*p* = 0.04). Most studies have shown possible relationships between chronic, systemic inflammatory responses caused by low serum albumin levels, impaired cellular immune response, and tumor cachexia [14,15,49,50,51]. We can speculate that the assessment of nutritional status and muscle mass of patients may predict the response to ICIs.

In our study, median OS (*p* = 0.005) and PFS (*p* = 0.06) were observed to be longer in smokers. In some studies, similar to our results, it has been reported that patients who are former/current smokers benefit more from ICIs compared to non-smokers [52,53]. Smoking-induced NSCLC is generally associated with elevated PD-L1 expression and elevated TMB levels [54]. We know that ICI treatment results in greater expression of neoantigens, which can enhance the anticancer immune response.

In our study, immune-related adverse events (irAEs) were noted in 56.7% of patients. Although dermatological toxicity was less than in the literature, hypothyroidism was found frequently. Instead of acute nausea, vomiting, and myelosuppressive effects which are frequently observed in cytotoxic chemotherapies, late-onset, inflammatory, or autoimmune-related adverse event profiles are more prominent in immunotherapies [55]. When patients with NSCLC receive immunotherapy for the first time, the body’s immune and inflammatory responses are most intense [56]. The main mechanism of the occurrence of irAEs is thought to be damage to their tissues due to the activation of lymphocytes reacting with their antigens following administration of ICIs [6]. The relationship between a lower NLR and higher incidence of irAEs has been reported previously [57]. In addition, the predictive value of NLR, LDH, and PNI on PFS, OS, or irAEs has been reported [58]. In 269 aNSCLC patients, the change in PIV at baseline and weeks 3–4 was shown to be associated with OS, PFS, and irAEs [48]. However, we could not show a relationship between the seven immune indexes and side effects. We thought that the number of patients with side effects and the fact that the number was further reduced when we grouped them within themselves may have affected our results. Especially in our patient groups who used nivolumab for a long time, we think that the longer the follow-up period, the greater the exposure, and accordingly the changes in the number of immune cells may affect the results. We hope that we will encounter different results in longer follow-up periods

The majority of our patients had ECOG PS 1, and at least one comorbid condition. A poor ECOG PS probably leads to less benefit from ICIs due to a frail immune system with reduced functional lymphocytes and a shorter life expectancy. Therefore, patients with a poor ECOG PS are usually excluded from ICI trials and are also underrepresented in studies designed for special populations that are not usually included in clinical trials [59]. In our study, patients who received at least four administrations of nivolumab and who underwent at least one response assessment were included. The proportion of patients with ECOG PS 2 was lower. Due to the lack of general health insurance reimbursement for ICI treatments in the first line in our country, our study included patients who received nivolumab only in the second line and beyond.

Our study was a single-center study and laboratory tests and imaging methods were performed in the same center. All consecutive NSCLC patients treated with nivolumab were entered into the study, limiting potential selection bias. We know that the true values are heterogeneous and the rates of variation increase between different centers. The single-center nature of our study was an advantage in terms of more homogeneity in terms of laboratory parameters and patient responses. To reduce the effect of changes on laboratory values, we included only laboratory data collected in the last week before starting nivolumab. Secondly, PD-L1 IHC status was only available in a fraction of patients as it was not routinely assessed during treatment.

There are some limitations of our study. The first is the retrospective design of the analysis, which may have revealed potential biases and confounding factors, and the importance of subgroup analysis based on the small sample size is limited. Many clinical indicators, concomitant diseases, complications, and even the process of processing clinical specimens can affect the serum concentration of each index. Standardized cut-off values for inflammation indexes in peripheral blood are still not available. The cut-off values we obtained may not be valid for all patients and should be confirmed in a large sample size, excluding further confounding factors. Even a single baseline indicator may contain errors. Cancer is a progressive disease, and we think that inflammatory and immune status can be evaluated more accurately with not only initial parameters but also parameters performed at regular intervals. In addition, the inevitable variation in salvage therapies may have positively or negatively affected the results of each group. An additional drawback of our study is the lack of a control arm, which leads to the conclusion that all these indexes are prognostic rather than predictive for immunotherapy.

We determined that seven immune indexes, along with NLR and d-NLR, which are the main specific parameters of the balance between immunity and inflammation, have specific clinical value in the immunotherapy treatment responses of patients with NSCLC receiving immunotherapy. We found that PIV, SII, PLR, and NLR are effective in long-term response to treatment, while the d-NLR immune index can guide us in patients with short-term progression. The relationship between the PNI immune score and very good response to ICI treatment supported the relationship between immunity–nutrition and treatment responses with real-life data. The high immune indexes in squamous histology (especially NRL, d-NLR, PLR, and PIV) suggest that the relationship between histological types and the immune system requires a clearer explanation. 

In conclusion, in this era of rapidly increasing cancer incidence, these simple and easily accessible indexes will help to predict treatment response in crowded oncology clinics. Larger-scale, longer-term, and more homogenous studies are needed to determine which index is more efficient in estimating the level of systemic inflammation and prognosis in specific cancer types. The mechanisms underlying inflammatory indexes in peripheral blood to determine the efficacy and outcomes of immunotherapy remain unclear. We need new models that combine clinical data with ongoing radiomics and proteogenomic studies, facilitating rapid clinical decision-making. A novel immune–nutritional index, including five parameters (i.e., neutrophils, monocytes, platelets, lymphocytes, and albumin) in addition to other predictive biomarkers, may contribute to the prognostic value in NSCLC patients receiving treatment with ICIs.

## Figures and Tables

**Table 1:** Baseline characteristics of patients.

Variable	Number of Patients (N, %)	Median (Min–Max)
Age (years)		62.0 (39–82)
<65	71 (68.3)	
≥65	33 (31.7)	
Gender		
Male	94 (90.4)	
Female	10 (9.6)	
BSA (median)		1.79 (1.5–2.16)
BMI (median)		24.2 (15.4–38.1)
ECOG PS		
0	41 (38.4)	
1	58 (55.8)	
2	5 (4.8)	
Smoking status		
Non-smoker	5 (4.8)	
Former smoker	43 (41.4)	
Current	56 (54.8)	
Comorbidity		
Present	64 (61.5)	
Absent	40 (38.5)	
PD-L1 IHC status		
Positive	24 (23.1)	
Negative	48 (46.2)	
Unknown	32 (30.8)	
Histology		
Squamous cell carcinoma	48 (46.2)	
Non-squamous cell carcinoma	56 (53.8)	
Location		
Right lung	65 (62.5)	
Left lung	39 (37.5)	
Metastasis at diagnosis		
Non-metastasis	12 (11.5)	
De novo metastasis	92 (88.5)	
Location of metastasis		
Lung parenchyma	18 (17.3)	
Liver	8 (7.7)	
Bone	33 (31.7)	
Adrenal gland	11 (10.6)	
Brain	9 (8.7)	
Other	29 (27.9)	
Brain metastasis		
No	78 (75.0)	
Solitary	17 (16.3)	
Oligo metastasis	3 (2.9)	
Multiple	5 (4.8)	
Type of response (first line treatment)		
Complete response	3 (2.9)	
Partial response	61 (58.7)	
Stable response	9 (8.7)	
Progressive disease	29 (27.9)	
Line of Nivolumab treatment		
2	90 (86.6)	
3	7 (6.7)	
4	7 (6.7)	
Type of response (Nivolumab treatment)		
Complete response	5 (4.8)	
Partial response	37 (35.6)	
Stable response	19 (18.3)	
Progressive disease	35 (33.7)	
PIV (median)		682.9 (7.79–5257.05)
SII (median)		848.57 (9.16–11485.03)
NLR (median)		3.08 (0.04–24.13)
PLR (median)		178.73 (1.00–1220.51)
MLR (median)		0.46 (0.01–1.64)
d-NLR (median)		1. 86 (2.65–6.35)
PNI (median)		40.22 (26.44–48.42)

Abbreviations: BSA, body surface area; BMI, body mass index; ECOG PS, Eastern Cooperative Oncology Group performance status; PD-L1, programmed death ligand 1; NLR, neutrophil-to-lymphocyte Ratio; PLR, platelet-to-lymphocyte ratio; MLR, monocyte-to-lymphocyte ratio; d-NLR, derived neutrophil-to-lymphocyte ratio; SII, systemic immune inflammation index; PIV, pan-immune inflammation value; PNI, prognostic nutritional index.

**Table 2:** Univariate Cox proportional hazard models for PFS and OS.

Variable	PFS			OS		
	HR	Univariate 95% CI	*p*	HR	Univariate 95% CI	*p*
Age						
<65	Ref			Ref		
≥65	0.46	0.14–1.47	0.19	2.41	0.57–10.18	0.23
ECOG PS						
0	Ref			Ref		
1–2	1.73	0.73–4.10	0.21	0.38	0.13–1.11	0.07
Smoking status						
Non-smoker/Former smoker	Ref			Ref		
Current	0.67	0.27–1.65	0.39	0.21	0.71–0.62	0.005
BMI						
<25	Ref			Ref		
≥25	2.44	0.92–6.34	0.07	0.29	0.07–1.13	0.07
Comorbidity						
Absent	Ref			Ref		
Present	2.73	1.10–6.81	0.03	0.57	0.14–2.22	0.41
Histology						
Non-squamous	Ref			Ref		
Squamous	0.50	0.20–1.29	0.15	0.81	0.20–3.20	0.76
PDL-1						
Negative	Ref			Ref		
Positive	1.07	0.47–2.47	0.85	0.59	0.21–1.61	0.30
Metastasis at diagnosis						
Non-metastasis	Ref			Ref		
De novo metastasis	1.27	0.23–6.84	0.77	11.67	0.85–159.57	0.06
Median number of cycles of nivolumab treatment						
≤8	Ref			Ref		
8<	0.02	0.00–0.12	0.0001	0.98	0.22–4.28	0.98
PIV						
Low	Ref			Ref		
High	1.14	0.14–8.88	0.90	2.88	0.38–21.52	0.30
SII						
Low	Ref			Ref		
High	0.66	0.07–6.15	0.72	0.26	0.02–3.31	0.30
NLR						
Low	Ref			Ref		
High	0.96	0.27–3.36	0.96	2.87	0.74–11.15	0.12
PLR						
Low	Ref			Ref		
High	1.43	0.44–4.68	0.54	0.49	0.08–2.99	0.44
MLR						
Low	Ref			Ref		
High	1.58	0.58–4.30	0.36	2.08	0.56–7.75	0.27
d-NLR						
Low	Ref			Ref		
High	1.78	0.45–7.04	0.40	4.89	0.98–24.20	0.05
PNI						
Low	Ref			Ref		
High	1.00	0.37–2.71	0.98	0.76	0.22–2.59	0.66

Abbreviations: BMI, body mass index; ECOG PS, Eastern Cooperative Oncology Group performance status; PD-L1, programmed death ligand 1; NLR, neutrophil-to-lymphocyte ratio; PLR, platelet-to-lymphocyte ratio; MLR, monocyte-to-lymphocyte ratio; d-NLR, derived neutrophil-to-lymphocyte ratio; SII, systemic immune inflammation index; PIV, pan-immune inflammation value; PNI, prognostic nutritional index.

## Data Availability

Data are contained within the article.
